# Possible Contribution of Meaning in Life in Patients With Chronic Pain and Suicidal Ideation: Observational Study

**DOI:** 10.2196/35194

**Published:** 2022-06-13

**Authors:** Vasileios Chytas, Alessandra Costanza, Viridiana Mazzola, Christophe Luthy, Vasiliki Galani, Guido Bondolfi, Christine Cedraschi

**Affiliations:** 1 Department of Psychiatry Service of Liaison Psychiatry and Crisis Intervention Geneva University Hospitals Geneva Switzerland; 2 Department of Psychiatry Faculty of Medicine University of Geneva Geneva Switzerland; 3 Faculty of Biomedical Sciences Università della Svizzera Italiana Lugano Switzerland; 4 Division of General Medical Rehabilitation Geneva University Hospitals Geneva Switzerland; 5 Department of Geriatrics and Rehabilitation Faculty of Medicine University of Geneva Geneva Switzerland; 6 Division of Clinical Pharmacology and Toxicology Multidisciplinary Pain Centre Geneva University Hospitals Geneva Switzerland

**Keywords:** meaning in life, suicidal ideation, chronic pain, pain, suicide

## Abstract

**Background:**

Chronic pain is associated with an elevated risk of suicidal ideation (SI).

**Objective:**

We aimed to examine if the presence or the search for Meaning in Life (MiL) are associated with less SI and explore whether MiL profiles emerge in our cohort. These profiles can be described as high presence–high search, high presence–low search, low presence–low search, and low presence–high search.

**Methods:**

In this observational study, we recruited 70 patients who were referred to the Multidisciplinary Pain Center of the Geneva University Hospitals and who answered positively to question 9 on the Beck Depression Inventory, 2nd Edition, investigating SI. Patients who agreed to participate in the study were further investigated; they participated in a structured diagnostic interview to screen for psychiatric diagnoses. During this interview, they completed the Meaning in Life Questionnaire and the semistructured Scale for Suicide Ideation (SSI) to assess the characteristics and severity of SI.

**Results:**

There was a statistically significant correlation between the presence of MiL subscale and the SSI. These 2 scales had a negative and statistically highly significant correlation (*R*=–.667; *P*<.001). The results also showed a negative and statistically highly significant correlation between the score of the search for MiL and the SSI (*R*=–.456; *P*<.001). The results thus pointed to the presence of MiL as a potential protective factor against the severity of SI, while the search for MiL is also a possible resiliency factor, although to a lesser extent. The profile low presence–low search grouped the vast majority (47%) of the patients; in these patients, the mean SSI score was 14.36 (SD 5.86), much higher compared with that of the other subgroups.

**Conclusions:**

This study’s results point to MiL as a concept of interest regarding devising psychotherapeutic interventions for chronic pain patients in order to reduce the suicidal risk and more accurately determine patients’ suffering.

## Introduction

Patients suffering from chronic pain conditions are at an elevated risk of suicidal behavior (SB). The literature points to a 20% to 40% prevalence rate of suicidal ideation (SI); lifetime prevalence of suicidal attempts (SA) is estimated between 5% and 14%; further, the risk of completed suicide is doubled in patients with chronic pain as compared to controls [1-4]. In most studies, the associations between chronic pain and SB were robust even after adjusting for the effect of sociodemographic characteristics and psychiatric comorbidities, including depressive conditions [5-11]. Numerous specific conditions that can modulate the SB risk in patients with chronic pain have been investigated (eg, pain characteristics, functional interference, illness beliefs, access to opioids) [1,3,12].

However, the literature also highlights the need for further exploration of other possible risk and protection factors to improve the characterization of the SB risk profile in these patients [3]. Using this framework, we will explore the role of meaning in life (MiL) as a resiliency factor possibly modulating SI in patients with chronic pain. Experiencing chronic pain or any other illness often requires revising one’s life goals and expectations [13]. Hence, its effect is not only limited to the patients’ biopsychosocial functioning, but it also affects the existential domain [14]. This common clinical observation raises the question of MiL in chronic pain patients with SI. A reduction of SI in patients attending an interdisciplinary treatment for chronic pain has been reported in approximately one-third of the participants [15]. A larger investigation of SB risk and resiliency factors in chronic pain, beyond the well-known demographic and psychiatric factors, has been strongly advocated for [3].

According to the conceptualization of MiL, this construct is a web of connections that help to read and understand the pain experience and create strategies directed at achieving the expected outcomes regarding reducing pain and its consequences [16]. This model divides MiL into 2 constructs, presence of and search for MiL [17], which are not mutually exclusive [18,19]. Many consider the presence of MiL as beneficial [20]; the search for MiL appears more controversial with some authors considering it the essence of human motivation [21] and others as a sign that one’s life has lost meaning [22] or has less meaning [23,24]. Different MiL profiles recently have been characterized in patients with chronic pain, resulting from the combination between low and high levels of presence and search for MiL, which were associated with a unique adjustment outcome: patients having profiles with high scores of presence showed fewer depressive symptoms and greater life satisfaction [14,25]. The literature does not yet mention the association of MiL with SI in patients with chronic pain.

Drawing on these studies and the MiL construct, this study aimed to investigate the relationship between MiL and intensity of SI in patients with chronic pain presenting SI.

Our objectives were as follows:

- Examine whether the presence of or search for MiL are associated with less SI
- Explore whether previously described MiL profiles (high presence–high search, high presence–low search, low presence–low search, and low presence–high search) emerge in our cohort

This study’s results will permit future exploration into further psychotherapeutic interventions integrating MiL profiles on the model of already existent standards for patients with other somatic conditions [26-29].

## Methods

### Setting and Procedure

The Division of Clinical Pharmacology and Toxicology and the Service of Liaison Psychiatry and Crisis Intervention at the Multidisciplinary Pain Center (MPC) of the Geneva University Hospitals conducted this observational study [30]. The MPC is a third-line ambulatory referral center to which treating physicians refer most patients for an interdisciplinary clinical evaluation and review of treatment proposals (eg, physical treatment, individual or group psychiatric/psychological treatment, or pharmacological proposals).

Participants were enlisted via ongoing recruitment through a project anchored in daily clinical practice at the MPC. Each participant received a series of self-administered screening questionnaires at home before the first routine visit to the MPC. All patients received these questionnaires regardless of their participation in this study because these questionnaires are routinely used at the MPC and they constitute an important clinical and professional tool. These questionnaires included the Beck Depression Inventory, 2nd Edition (BDI-II) [31]. In a second step, approximately 15 days after receiving the self-administered routine screening questionnaires, patients were seen at the MPC. During this consultation, patients with SI (ie, those who answered positively [responses 1, 2, and 3] to question 9 of the BDI-II) received written and oral information about this study from a member of the medical team who also responded to their questions. If serious suicidal thoughts were identified, all appropriate measures were taken, including, if necessary, accompanying patient to the psychiatric emergency ward at the Geneva University Hospitals. In a third and final step, 1 to 7 days after the visit to the MPC, patients with SI who agreed to participate in the study attended a second visit. During this consultation, patients signed informed consent and were included in the study. They also participated in a clinical interview and structured diagnostic interview, the French version 5.0.0 of the Mini-International Neuropsychiatric Interview (MINI) [32], to screen for psychiatric diagnoses according to the *Diagnostic and Statistical Manual of Mental Disorders, 4th Edition* (DSM-IV). During this same interview, patients completed the Meaning in Life Questionnaire (MiLQ) [23]. In addition, a clinical evaluation of SI was conducted as well as a semistructured scale, the Scale for Suicide Ideation (SSI), to assess the characteristics and severity of SI [33].

Inclusion criteria were patients with chronic pain, referred to the MPC, presenting with an SI, indicated by a positive response (responses 1 to 3) to question 9 of the BDI-II, aged over 18 years, and providing written informed consent. Exclusion criteria were insufficient understanding of the French language, diagnosis of organic mental disorders (F00-F09), psychotic disorders (F20-F29), or borderline personality disorder (F60.3).

### Instruments

We used the instruments presented in Table 1.

**Table 1:** Instruments used for the assessment.

Instruments	Main characteristics of the instruments
Question 9 of the Beck Depression Inventory [31]	Self-report multiple choice inventory Indicator of the severity of depressive symptoms; standard cutoff scores: 0-10 (no depression), 11-19 (mild depression), 20-29 (moderate depression), >30 (severe depression) 21-items, each rated on a 4-point scale ranging from 0-3 based on severity of the item Question 9: “Suicidal thoughts or wishes” (0: I don’t have any thoughts of killing myself; 1: I have thoughts of killing myself, but I would not carry them out; 2: I would like to kill myself; 3: I would kill myself if I had the chance).
Scale for Suicide Ideation [33,34]	Scale based on a semistructured interview with the patient Indicator of characteristics/severity of an individual’s plans and wishes to commit suicide 19 items, each rated on a 3-point scale ranging from 0-3 based on severity of the item Total for the 19 items: minimum=0, maximum=38 (higher scores indicate greater SI^a^); score ≥6 indicates clinically significant SI [35,36]
Meaning in Life Questionnaire [23]	Measure of presence of and search for (5 questions each) MiL^b^ For each subscale, scores range from 5-35, high scores indicating high presence of or search for MiL. MiLQ^c^ does not have definitive cutoff scores as it measures MiL across the range of human functioning; the author provides probabilistic estimates about scores above, equal, or below 24 on presence of and search for constructs [37].

^a^SI: suicidal ideation.

^b^MiL: meaning in life.

^c^MiLQ: Meaning in Life Questionnaire.

The BDI-II [31] has been repeatedly used in the context of chronic pain [38,39] and has undergone extensive validation, including in the French language [40,41]. The importance of somatic symptoms of depression in pain patients has been stressed along with the risk of a score inflation in people with chronic pain [42] and hence the importance of using a structured clinician-administered interview to reliably identify a possible depressive disorder [43].

Considering the BDI-II screens depressive symptoms and is not a diagnostic tool, an experienced psychiatrist also conducted an interview using the MINI, which is extensively used to screen for probable psychiatric diagnoses according to the DSM-IV and ascertain the presence of depression in various somatic or psychiatric contexts. The French version has been used [44].

The SSI [33,34] assesses the characteristics and severity of SI. This interviewer-administered scale is one of the most widely used instruments for assessing suicidal thinking. It helps to identify suicidal individuals if they are willing to acknowledge and share their thoughts. The SSI serves as a routine screening for existent suicidal thinking and can aid in an exploration that is more extensive regarding the severity of such thoughts. It can be administered in various settings (eg, general medical services, psychiatric services) and during a routine screening [33]. The SSI has a validated French version [45,46] that has already been used in the context of the Geneva University Hospitals in Switzerland [47].

The MiLQ [23] has 2 subscales: presence of and search for MiL. The MiLQ has been translated into French [47] and used in the context of the Geneva University Hospitals in Switzerland [47,48]. Other researchers have used it with patients suffering from chronic pain [25], depression [49], or other chronic health problems [14]. We further used the MiLQ to define the profiles of MiL (high presence–high search, high presence–low search, low presence–low search, and low presence–search high) as other authors suggested and used the same method of analysis on the results obtained in our sample [14].

### Statistical Analyses

Data from all questionnaires were manually entered into an SPSS database (IBM Corp). Double data entry was performed to minimize entry errors. Questionnaires and demographic data were analyzed with SPSS 22. Descriptive statistical analyses were performed to calculate the means and standard deviations of the numeric variables and the frequencies and percentages of the categorical variables. The chi-square test was used for categorical variables and to check for correspondence between groups with respect to age, sex, and years of education. To test our main hypothesis, a nonparametric statistical dependence measure (Spearman rho) or a Pearson correlation coefficient was calculated to look for any significant interaction between the MiLQ subscales (ie, presence of or search for) and SI. As for missing values, the analysis was performed across all questionnaire scores to look for significant trends. Random missing values were replaced with the mean.

### Ethics Approval

The Ethics Committee of the Canton of Geneva approved the protocol (project No. 2017-02138; decision dated January 25, 2018) that has been carried out in accordance with the research plan and Swiss legal and regulatory requirements, which are in agreement with the principles stated in the current version of the Declaration of Helsinki. We obtained written informed consent from all patients.

## Results

### Participants

A total of 70 patients with SI (ie, patients who answered >1 to question 9 of the BDI-II) were recruited. These patients were enlisted for 20 months between March 2018 and November 2019; 8 patients did not wish to participate in the study and another 4 were excluded because they did not have a sufficient mastery of the French language. These 82 patients who were initially contacted to participate in our study consist of about 15% of the total number of patients who were referred to the MPC during this period.

Table 2 presents the sociodemographic and clinical characteristics of the 70 patients who agreed to participate in the study. Of these, 60% (42/70) were women, had a mean age of 54 years, and were mostly professionally qualified. Only 14% (10/70) of patients were currently employed; 77% (54/70) were on sick leave due to pain, partial or full time, and among those, only 15% (11/54) of patients were engaged in professional reintegration measures.

### Clinical Characteristics

The origin of the pain was neuropathic or nociceptive in most of the patients. They had a pain history that was of long duration, with an average of 8 years, but also of high intensity, with an average intensity of current pain close to 7 out of 10 on a 100-mm visual analog scale (VAS), and as many as one-third of the patients (23/70, 32.9%) assessing the maximum intensity of their pain at 10 out of 10.

**Table 2:** Sociodemographic and clinical characteristics of the participants.

		Values
**Gender, n (%)**		
	Male	28 (40)
	Female	42 (60)
Age (years), mean (SD)		54.26 (14.5)
**Civil status, n (%)**		
	Married	31 (44)
	Single	13 (19)
	Separated/divorced	21 (30)
	Widowed	5 (7)
French as original language, n (%)		42 (60)
**Education, n (%)**		
	Primary school	13 (19)
	Apprenticeship	27 (38)
	Vocational school	21 (30)
	University	9 (13)
Present professional activity, n (%)		10 (14)
Pain duration (years), mean (SD)		8.1 (8.1)
**Pain intensity, mean (SD)**		
	VAS^a^ current	6.9 (2.4)
	VAS minimum	5.3 (2.8)
	VAS maximum	8.9 (1.4)
**Origin of pain, n (%)**		
	Neuropathic	44 (63)
	Nociceptive	22 (31)
	Visceral or other	4 (6)

^a^VAS: visual analog scale.

Most of the participants considered their pain as related to a specific event. This event was an accident in 30% (21/70), one or several surgeries or treatments were perceived as harmful in 15% (11/70), and emotional factors and traumatic life events occurred in another 12% (9/70). Some (17/70, 25%) of the patients attributed their pain to a disease and 10% (7/70) to heavy labor. Only about 8% (5/70) of the patients could not think of a specific cause.

### Depressive Symptomatology

The mean score of the BDI-II was 31.26 (SD 11.37). More precisely, the absence of depressive symptoms (BDI-II ≤10) was rare in our sample (2/70, 3%). On the other end of the spectrum, as many as 49% (34/70) of patients had a BDI-II score over 30, indicating symptomatology present during a severe depressive episode. Regarding the other patients, 14% (10/70) had a score between 11 and 20 (corresponding to a mild depressive episode) and 34% (24/70) had a score between 21 and 30 (depressive symptoms compatible with a moderate depressive episode).

The MINI structured clinical interview confirmed the presence of a depressive episode in the majority of these patients (68/70, 97%) and highlighted other psychiatric comorbidities among them, in particular anxiety disorders (panic disorder, social phobia, agoraphobia, and obsessive and compulsive disorder), which were largely represented (25/70, 36%).

### MiL and SI

The mean score on the SSI was 11.40 (SD 5.92). As a reminder, high scores on this scale indicate high SI, and the score of 6 is used as a cutoff to indicate clinically significant SI. In this group, 83% (58/70) of patients had an SSI score ≥6 and 17% (12/70) had a score of <6.

In our group, there was no significant correlation between the presence of or search for MiL and the intensity or duration of pain. Gender, origin of pain, marital status, or age also seemed not to affect the presence of or search for MiL. However, we found a significant difference in the presence of MiL between working and nonworking patients (*P*=.04) but not in the search for MiL.

We also investigated the relationship between MiL and SI, considering separately the 2 concepts, presence of and search for MiL. For each subscale, the scores vary between 5 and 35, with high scores signifying a high presence of or search for MiL. The cutoff for these 2 subscales is 24, with scores equal or higher indicating higher presence of or search of MiL. Regarding the presence of MiL, the average score on the presence of MiL subscale was 20.13 (SD 8.23). Among the patients in the study, 37% (26/70) presented a score for the presence of MiL ≥24; for 63% (44/70) the score was <24. Regarding the search for MiL, the average score on the subscale was 18.14 (SD 8.64); 30% (21/70) of patients had a score ≥24, and 70% (49/70) had a score of <24. There was also a positive and significant correlation between the presence of and the search for MiL (*R*=.402; *P*=.001).

Furthermore, there was a statistically significant correlation between the presence of MiL subscale and the SSI. These 2 scales had a negative and statistically highly significant correlation (*R*=–.667; *P*<.001). The results also showed a negative and statistically highly significant correlation between the score of the search for MiL and the SSI (*R*=–.456; *P*<.001).

Considering the results of the MiLQ in the 2 subgroups (ie, patients who presented a high SI [SSI ≥6] and those presenting lower SI [SSI <6]) highlighted the following results: in the first subgroup (SSI ≥6), the negative correlation between the severity of SI and the presence of MiL was statistically very significant (*R*=–.544; *P*<.001). This group also had an average score on the MiL presence subscale (18.19 [SD 7.35]) that was lower than the average score of the whole group (20.13). In contrast, in patients with a lower score on the SSI (SSI<6), the correlation between the presence of MiL and SI was negative but not significant and the mean score on the MiL presence subscale, considerably higher, was 29.50 (SD 5.44), above the mean score for the whole group.

The results were more contrasted for the search for MiL in these 2 subgroups. In the subgroup of patients with higher SI (SSI≥6), the mean score for the search for MiL was 17.52 (SD 8.84), close to the mean score of the whole group (18.14). In this subgroup, there was a strong and significant negative correlation between the search for MiL and SI (*R*=–.492; *P*<.001). In the subgroup of patients with a weaker SI (SSI<6), the mean score for the search for MiL was 21.17 (SD 7.17), higher than the mean score of the whole group (18.14). However, in this subgroup, the correlation between the search for MiL and SI was not significant.

Finally, we found a statistically significant difference concerning the presence of MiL subscale’s score between the 2 subgroups, patients with higher SI (SSI≥6) and those with lower SI (SSI<6), with *P*<.001. However, regarding the subscale of the search for MiL, the difference between these 2 subgroups was not significant.

### Profiles of MiL

We examined the relationship of the different profiles of MiL with SI, using the grouping Dezutter et al [14] described (ie, applying the same analysis to the scores obtained in our sample). In our study, 23% (16/70) of patients corresponded to the profile high presence–low search; those patients had an average score on the SSI of 8.56 (SD 5.11). On the other end, 16% (11/70) of patients who corresponded to the profile low presence–high search had an average score on the SSI of 10.45 (SD 4.30). The profile high presence–high search grouped 14% (10/70) of patients with an average SSI score of 7.20 (SD 3.99). The profile low presence–low search grouped the majority of the patients (33/70, 47%); in these patients, the mean SSI score was 14.36 (SD 5.86), which was much higher than that of the other subgroups.

## Discussion

### Principal Findings

This study showed that MiL is associated with SI in patients with chronic pain. Our results showed that the presence of MiL seemed to be a potentially protective factor against SI. The statistically significant negative association between the subscale presence of MiL and SSI, with the presence of MiL actually associated with a decrease in the severity of SI, further supports our results. We found this negative correlation both in the patients with a high SI (SSI ≥6) and in those who presented a lower SI (SSI <6). These results are in line with those of the literature, which highlight this negative correlation between the presence of MiL and SI among students [50], military and veterans, [28] and HIV-positive patients [51]. These data thus support our initial hypothesis, which held that the more a patient identifies meaning in their life, the lower the severity of their SI.

The results are less straightforward regarding the search for MiL. In our sample, we found a negative and significant correlation between the search for MiL and SI. This means that the patients who seek MiL, regardless of the presence of MiL, have less intense SI as compared to those who do not. Furthermore, this significant negative correlation is also found in the subgroup of patients who present with high SI (SSI ≥6), but this association is not significant in patients with less intense SI (SSI <6).

Contrary to the results for the presence of MiL, the difference in the scores on the search for MiL subscale is not significant between patients with strong SI (SSI ≥6) and patients with less intense SI (SSI <6). This shows that while the presence of MiL is clearly higher in patients with lower SI, the search for MiL tends not to be significantly different between patients with high versus low SI. Previous studies have also found a protective effect of searching for MiL against SI [52], but other studies have reported a positive correlation between searching for MiL and SA [28]. Overall, our results support the search for MiL as a factor that can protect against an increased severity of SI; however, less significantly as compared to presence of MiL.

In our group, neither presence of nor search for MiL were associated with sociodemographic or pain-related characteristics, except for professional activity, which was associated with the presence of MiL. This is also in line with previous research suggesting that patients who present MiL display a higher level of adaptation, including in the professional field [24]. Fewer working individuals seeking disability may mediate these results, as well as less MiL related to the professional sphere in these participants. This issue may be worth addressing in future studies.

Four profiles of MiL (high presence–low search, high presence–high search, low presence–high search, and low presence–low search) have been described in chronic pain patients [14]. The patients in the high presence–high search for MiL subgroup had a much lower SSI score compared to the other subgroups, followed by the high presence–low search subgroup.

The literature has reported that when the search for MiL is high and associated with a high presence of MiL, the determining effect of the search for meaning appears to decrease [14]. Therefore, in these patients, we believe that because they consider their life as having meaning, the additional search for meaning represents a healthy approach and that they thus present a good level of well-being and acceptance [14]. On the other hand, what differentiates our study from that of the Dezutter et al study [14] is that the first subgroup, high presence–high search for MiL, has fewer suicidal thoughts compared to the second, high presence–low search. Our results suggest that the search for meaning also plays its probable protective part against SI. This may be at least partly related to these patients’ expectations of the pain consultation as a means to relieve their pain and improve their quality of life while simultaneously seeking to give them meaning. Therefore, we see in our study in a more pronounced way an increased protective role of research as a lever of vital impetus in the patients who contact our center.

However, the search for MiL when no meaning is presently identified is associated with higher SI scores, thus pointing to the search for meaning as more anxiety provoking and stressful. Some have suggested that such a pattern might result in an adaption that is more problematic and less optimal psychological well-being [14]. In our study, we also identified patients corresponding to the profile high search–low presence of MiL and presenting with a higher average score on the SI scale when compared to the first 2 subgroups. Furthermore, we found a significantly higher SI score in patients who neither present nor search for an MiL. These patients constitute the group with the lowest level of adaptation and lowest well-being in the Dezutter et al [14] study, which, as we have done, examined the link between chronic pain and MiL. However, these results contrast with those of Steger et al [18], which suggests that for patients who fail to identify meaning in their life, seeking a meaning leads to a level of adaptation and to a particularly low well-being, even more than patients who neither present nor search for a meaning. In this case, the research shows it having an anxiety-inducing, even deleterious role. In the context of varying adaptation levels, the issue of psychological flexibility is of interest. Psychological flexibility has indeed been described as a key feature in the psychological approaches to chronic pain management [53] and has been largely used in the protocol called acceptance and commitment therapy (ACT). ACT treatment programs have received attention not only in the field of chronic pain but also as a means of reducing SI [54]. Hence, considering MiL and reducing SI as possible targets of ACT for chronic pain warrants further investigation.

Finally, our data confirmed that the presence of and the search for MiL are positively and significantly correlated, which suggests that the 2 constructs may depend on one another. This is not in line with previous research [23,24,47], but the role of intervening variables, acting as mediating factors, has not been explored in this study and clearly warrants further investigation.

### Limitations

Our study has limitations that need acknowledgment. First, the study sample is small, and thus not allowing for the construction of subgroups using the participants’ sociodemographic and clinical characteristics; however, the sample allowed investigating and categorizing the patients according to the MiL and SI dimensions. Second, the MiLQ has not been extensively validated in French; however, it has been used in various contexts, particularly psychiatric emergencies, in its current version [47]. Third, and importantly, the participants recruited in this study were only those who self-reported (ie, admitted) SI, and yet admitting SI has been described as highly problematic [55,56]. Indeed, both underreporting as a means of concealing symptoms [3,57] and overreporting as a means of soliciting increased attention or making a plea for help [58] may induce response bias. Patients who underreported SI may be underrepresented while those who may be prone to overreport may in turn be overrepresented, thus limiting the findings’ generalizability. Self-report screening measures have inherent limitations regarding eliciting the individuals’ phenomenological experiences but also knowing whether they provide an accurate report of the clinical phenomenon under investigation [59,60]. Fourth, this is a cross-sectional study, and this design does thus not allow for any inferences regarding a causal relationship between changes in MiL as leading to a reduction in SI. Taken together, these limitations also point to the existence of biases and possible confounders as one of the key methodological issues of correlational studies [61].

### Conclusion

Taken together, our results regarding the different MiL profiles and associated risk factors highlight the place to be given to individual participants. Indeed, MiL profiles may allow clinicians to consider mental and somatic suffering; however, the search for the coherence of a constellation (eg, pain status, functional interference, illness beliefs, emotional context, SI, and SA) should not mask the specific form of the individual patient’s suffering. Using MiL profiles can help clinicians recognize their patient’s suffering while keeping in mind the necessity to consider the patient’s singularity even while using validated tools to describe mental states and organization. In chronic pain patients, the continuity and attention of clinicians to maintain the associative work of the patient’s discourse is of main concern; indeed, facing suicidal crisis, the process at work in the patients can hinder this work. Considering the concept of MiL should support clinical work aimed at providing the most adequate response to patient suffering, as it has proven possible in the context of psychiatric emergencies [47,62]. Psychotherapeutic interventions targeting MiL have been found effective in reducing suicide risk [26] and represent a promising therapeutic opportunity [27-29] in various clinical contexts. However, this construct’s contribution to decreasing suicide risk in patients with chronic pain remains to be established. Our study may help find further tools to treat suffering and emotional distress in chronic pain patients through defining a unique and individualized suicide risk profile, improving screening and preventive strategies, and developing intervention strategies including the existential sphere. Clinical intervention research would be beneficial in determining if interventions based on active bolstering of MiL, such as methods of linking listening to the patients and understanding their phenomenological experience with strategies for increasing MiL, can affect outcomes for individuals presenting with SI and SB. The Attempted Suicide Short Intervention Protocol is such a treatment method to consider for exploring the patient’s experience and the follow-up [63-66].

Our study supports a probable protective role of MiL against the severity of SI in patients with chronic pain presenting with SI. Further studies should be conducted to better enlighten the complex relationship between suicidal risk, prevention, and intervention strategies in chronic pain patients to provide new perspectives. The results stress the importance of thorough patient interviews regarding bringing theoretical conceptualizations into daily clinical practice.

## Abbreviations

ACT: acceptance and commitment therapy
BDI-II: Beck Depression Inventory, 2nd Edition
DSM-IV: Diagnostic and Statistical Manual of Mental Disorders, 4th Edition
MiL: meaning in life
MiLQ: Meaning in Life Questionnaire
MINI: Mini-International Neuropsychiatric Interview
MPC: Multidisciplinary Pain Center
SA: suicidal attempt
SB: suicidal behavior
SI: suicidal ideation
SSI: Scale for Suicide Ideation
VAS: visual analog scale
